# Effects of α5 GABA_A_ receptor modulation on social interaction, memory, and neuroinflammation in a mouse model of Alzheimer's disease

**DOI:** 10.1111/cns.13914

**Published:** 2022-07-13

**Authors:** Jovana Aranđelović, Anja Santrač, Bojan Batinić, Lidija Todorović, Vladimir Stevanović, Veera Venkata Naga Phani Babu Tiruveedhula, Dishary Sharmin, Farjana Rashid, Boban Stanojević, James M. Cook, Miroslav M. Savić

**Affiliations:** ^1^ Department of Pharmacology, Faculty of Pharmacy University of Belgrade Belgrade Serbia; ^2^ Department of Physiology, Faculty of Pharmacy University of Belgrade Belgrade Serbia; ^3^ Laboratory for Radiobiology and Molecular Genetics, Vinča Institute of Nuclear Sciences, National Institute of thе Republic of Serbia University of Belgrade Belgrade Serbia; ^4^ Department of Chemistry and Biochemistry, Milwaukee Institute for Drug Discovery University of Wisconsin‐Milwaukee Milwaukee Wisconsin USA; ^5^ Comprehensive Cancer Centre, Faculty of Life Sciences & Medicine, King's College London Rayne Institute London UK

**Keywords:** 5xFAD, Alzheimer's disease, cognition, neuroinflammation, α5 GABA_A_ receptor modulation

## Abstract

**Aims:**

GABAergic modulation involved in cognitive processing appears to be substantially changed in Alzheimer's disease (AD). In a widely used 5xFAD model of AD, we aimed to assess if negative and positive allosteric modulators of α5 GABA_A_ receptors (NAM and PAM, respectively) would affect social interaction, social, object and spatial memory, and neuroinflammation.

**Methods:**

After 10‐day treatment with PAM, NAM, or solvent, 6‐month‐old transgenic and non‐transgenic 5xFAD mice underwent testing in a behavioral battery. Gene expressions of IL‐1β, IL‐6, TNF‐α, GFAP, and IBA‐1 were determined in hippocampus and prefrontal cortex by qPCR.

**Results:**

PAM treatment impaired spatial learning in transgenic females compared to solvent‐treated transgenic females, and social recognition in transgenic and non‐transgenic males. NAM treatment declined social interaction in transgenic and non‐transgenic males, while had beneficial effect on cognitive flexibility in non‐transgenic males compared to solvent‐treated non‐transgenic males. Transgenic animals have not fully displayed cognitive symptoms, but neuroinflammation was confirmed. NAM reduced proinflammatory gene expressions in transgenic females and astrogliosis in transgenic males compared to pathological controls.

**Conclusion:**

PAM and NAM failed to exert favorable behavioral effects in transgenic animals. Suppression of neuroinflammation obtained with NAM calls for more studies with GABAergic ligands in amyloid beta‐ and/or tau‐dependent models with prominent neuroinflammation.

## Graphical abstract

MP‐III‐022 and PWZ‐029, as negative and positive allosteric modulators of α5 GABA_A_ receptors, respectively, exert negligible effects on social interaction, social, object, and spatial memory in 5xFAD mouse model of Alzheimer's disease. PWZ‐029 reduces proinflammatory gene expressions in transgenic females and astrogliosis in transgenic males, which justifies further studies with GABAergic ligands in models of Alzheimer's disease.

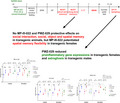

## 1 INTRODUCTION

Alzheimer's disease (AD) is a devastating neurological illness that predominantly affects cognition in elderly people, resulting in disability.^1^ In AD pathogenesis, the GABAergic system emerged as an important facet in cognitive impairment.^2^ GABA_A_ receptors containing the α5 subunit are common but not ubiquitous in the brain, with roles in neuronal excitability, synaptic plasticity, neurogenesis, and cognition in the hippocampus being mediated by both synaptic and tonic inhibition.^3^ Accumulating evidence supports the involvement in AD pathology of α5 GABA_A_ receptors in various brain subregions, most conspicuously the hippocampus and cerebral cortex.^4^

Excitatory/inhibitory imbalance, observed in AD patients, is likewise present in mouse transgenic models of AD with amyloid beta (Aβ) accumulation and cognitive decline.^3^ One such model is 5xFAD mouse model, characterized by an early onset of amyloid plaques deposition in cortical and hippocampal areas.^5^ It was suggested that excessive glutamatergic stimulation in an early stage of disease in 5xFAD model is probably compensated by higher GABA currents.^6^ The induced prolonged tonic inhibition could give rise to memory impairment, which was restored by acute administration of a negative allosteric modulator (NAM) of α5 GABA_A_ receptors (L‐655,708) or with SNAP‐5114 to block astrocytic GABA release via GAT3/4.^6^ Apparently contradictory, in another mouse model of AD it was shown that both, activation and inhibition of GABA_A_ receptors (by muscimol and bicuculline, respectively), may improve spatial recognition memory.^7^ In various tests other than those modeling AD, both a positive allosteric modulator (PAM)^8^, ^9^, ^10^ and NAM^11^, ^12^ appeared to have a potential to restore object and spatial memory, with PAM being favored in the context of aging.^8^

Activity of mouse parvalbumin‐positive (PV) interneurons in medial prefrontal cortex (PFC) elicited by low gamma frequency stimulation, as well as muscimol treatment in the dorsomedial PFC, resulted in a prosocial effect leading to increased social interaction.^13^ Gene expression study in PFC demonstrated existence of the α5 subunit in pyramidal neurons (39.7% of α5 GABA_A_ receptor positive cells in humans and 54.14% in mice) and in PV interneurons (20% in humans and 16.33% in mice).^14^ Positive allosteric modulation at α5 GABAA receptors showed improved social interaction in a rat autism model.^10^ On the contrary, both, pharmacogenetic inhibition of PV neurons^13^ and bicuculline stereotaxic administration in the PFC^15^ decreased social interaction. Even though a selective α5 GABA_A_ receptor NAM failed to decrease social interaction in healthy rats,^16^ FG 7142, as a non‐selective inverse agonist acting also at α5 GABA_A_ receptors,^17^ did reduce social interaction.^16^ Hence, one could posit that α5 GABA_A_ receptor PAM and NAM may mitigate and further deteriorate sociability impairment in AD, respectively.

Although Aβ and tau accumulations seemed to be a hallmark of AD for decades, even the most advanced and promising pharmacological strategies directed to reduce Aβ load failed so far to demonstrate consistent cognitive improvements in AD patients (cf. Ref. [18]). Thus, research focus becomes shifted to other contributing mechanisms, such as neuroinflammation.^19^ A predominant proinflammatory phenotype is observed in microglia exposed to Aβ in an in vitro AD model,^20^ as well as during the pre‐plaque stage in murine AD models.^21^ Such a proinflammatory microglial phenotype was also observed in humans with mild cognitive impairment without detected Aβ,^22^ which may suggest its protective role at the beginning of disease, with an opposite role when AD progresses.^19^ Activated microglia secrete proinflammatory cytokines, and could further induce an astrocytic shift to a proinflammatory phenotype.^23^ Neurotoxic reactive astrocytes were detected in post‐mortem brain tissue, and around 30%–60% of astrocytes in hippocampus and PFC in AD patients had a proinflammatory phenotype.^24^ Furthermore, proinflammatory astrocytes could cause excessive GABA release affecting memory in AD models,^25^ and also lead to neuronal and oligodendrocyte death.^19^

Based on the listed evidence, we aimed to assess the influence of protracted bidirectional α5 GABA_A_ receptor modulation on different cognitive domains and sociability in 6‐month‐old transgenic and non‐transgenic 5xFAD mice of both sexes, as well as on neuroimmune profile in the hippocampus and PFC, characterized by gene expression of proinflammatory cytokines IL‐1β, IL‐6 and TNF‐α, as well as of markers of microglial activation (ionized calcium‐binding adaptor molecule‐1 [Iba‐1]) and astrogliosis/astrocyte damage (glial fibrillary acidic protein [GFAP]).

## 2 METHODOLOGY

### 2.1 Substances

PWZ–029 ((methyl(8–chloro–5,6–dihydro–5–methyl–6–oxo–4H–imidazo[1,5–α][1,4]benzodiazepin–3–yl) methyl ether)) as a NAM of α5 GABA_A_ receptors,^26^ and MP–III–022 ((R)–8–ethynyl–6–(2–fluorophenyl)–N,4–dimethyl–4H–benzo[f]imidazo[1,5–a][1,4]diazepine–3–carboxamide) as a PAM of α5 GABA_A_ receptors (Stamenic et al., 2016)^27^, were used in this study. Selected substances, synthesized at the Department of Chemistry and Biochemistry, University of Wisconsin—Milwaukee, USA, were dissolved in solvent (SOL) prepared of 85% distilled water, 14% propylene glycol, and 1% Tween 80. Ligands were dosed at 5 mg/kg once per day during 10 days in transgenic and non‐transgenic animals, while control animals obtained SOL.

### 2.2 Animals and experimental design

Transgenic 5xFAD mice with five human mutations of APP and PSEN1: the Swedish (K670N/M671L), Florida (I716V), and London (V717I) mutations in APP, and the M146L and L286V mutations in PSEN1, and their non‐transgenic littermates were kept together in Plexiglas home cages in groups of 4–6 per cage with food and water ad libitum under 12:12 light–dark cycle (lights on at 06.00 h). The colony was kindly provided by the Institute for Biological Research “Siniša Stanković”, University of Belgrade, Belgrade, Serbia, and the animals were born and reared in the vivarium of Faculty of Pharmacy – University of Belgrade, Belgrade, Serbia.

Behavioral experiments were run in the light phase (from 07.00 to 17.00 h) and followed ARRIVE 2.0 guidelines (PMID: 32663096). The study was accomplished within the project approved by the Ethical Council for the Protection of Experimental Animals of the Ministry of Agriculture, Forestry and Water Management of the Republic of Serbia.

**FIGURE 1:**
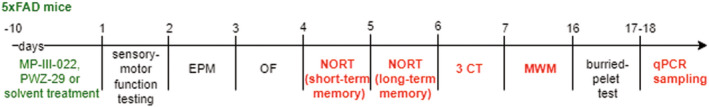
Timeline of behavioral battery conducted on each 5xFAD animal cohort treated with MP‐III‐022, PWZ‐029, or solvent. Red text signifies experiments analyzed and discussed in this manuscript, while tests without coloring have been already published.^28^ Abbreviations: 3 CT, three‐chamber test; EPM, elevated plus maze; MWM, Morris water maze; NORT, novel object recognition test; OF, open field

5xFAD transgenic and non‐transgenic animals of both sexes, reared and tested in three timely separated and consecutive subcohorts, randomly received i.p. treatment with MP‐III‐022, PWZ‐029 or SOL during 10 days at 6 months ±2 weeks of age. After completing the treatment protocol, each animal subcohort underwent a behavioral battery comprised of tests for sensory and motor abilities, elevated plus maze, open field (the results published in^28^), novel object recognition test, three‐chamber test, and Morris water maze test (Figure 1). Hence, all animals were subjected to the same experimental procedure under considerably similar conditions, but divided in three time points, which is relevant for increasing reproducibility of this study.^29^ Following behavioral procedures, animals were terminally anesthetized with ketamine (100 mg/kg, i.p.), PFC and hippocampus were collected and snap‐frozen in liquid nitrogen. Samples were stored at −80°C until qPCR analysis was conducted.

### 2.3 Behavioral tests

#### 2.3.1 Novel object recognition test

In a Plexiglas box (40 × 30 × 30 cm), test animals were exposed during 10 min to a pair of two identical objects located 10 cm far from each other (plastic Rubik's cube or cylinder of glass, randomly distributed across groups), according to the protocol adopted and modified from Colié et al.^30^ After 45 min, one of the objects was replaced by the remaining new object and test animal was allowed to freely explore them for 10 min (Novel object recognition test (NORT) for short‐term memory assessment; NORT short‐term). 24 h later, animals were again introduced to the same arena for 10 min, but the old object was replaced by the new one (plastic pyramid) (NORT for long‐term memory assessment; NORT long‐term).

#### 2.3.2 Three‐chamber test (3ct)

Non‐transparent Plexiglas apparatus consisted of three parts: the center chamber and two identical side chambers, as previously described.^31^ The test comprised of three consecutive phases: habituation, social interaction test (SIT), and social recognition test (SRT). The duration of the first phase was 5 min, and test mouse was allowed only to explore the center zone, with entries to side chambers closed. In the SIT phase, the test mouse was placed into the center chamber, with the passages blocked and a single unfamiliar mouse was placed into either of the side chambers under the wire cage, while the cage in another chamber remained empty. The sliding doors were opened, and the test mouse was allowed to move freely within the apparatus for 10 min. The test mouse was then returned to the center chamber, the doors were closed, and a new unfamiliar mouse was enclosed into the empty cage in the other side chamber. Next, the SRT phase began with opening the sliding doors and the test mouse was allowed to move freely within the apparatus for another 10 min. Test animal exploration time in the narrow zone of cage with or without stimulus animal was used in statistical analysis.

#### 2.3.3 Morris water maze

Animals were tested in a large tank (170 cm diameter), and the protocol consisted of two main phases: regular and reversal phase. Regular phase began with the cued‐platform task, and animals were allowed to locate the cued platform in the pool. From days 2 to 5, animals were trained to find the hidden platform. At day 6, in the probe trial, the platform was removed, and animals swam only once for 1 min from the position most distant to the platform zone. On the first day of the reversal phase (reversal cued day), a new platform was positioned in the opposite quadrant compared to the old platform. On the following day (reversal training day), the platform was hidden. In the reversal 1‐min probe trial, animals were let to explore the maze starting from the position farthest to the removed new platform.

With the exception of the probe swims, trial duration in all phases of MWM was 2 min, and an animal was allowed to swim in 4 consecutive trials per day with pseudorandom departure points. If the animal was unable to reach the platform, it was guided to the platform and allowed to remain there for 25 s. The average of 4 consecutive trials in the regular training phase was calculated and used for statistical analysis. In reversal cued, latencies to locate the new platform in the first two trials per test day, labeled as trial 1 and trial 2, respectively, were analyzed as modified from Hu et al.^32^

The latency to find the platform and number of entries in the platform zone, and old and new platform and number of entries in these platform zones in regular probe and reversal probe, respectively, were used for statistical analysis.

### 2.4 Tissue preparation and qPCR

Total RNA was isolated by Trizol protocol and RNA concentration was measured. After reverse transcription reaction, the converted cDNA underwent real‐time polymerase chain reaction (qPCR) in order to determine IL‐1β, IL‐6, TNF‐α, GFAP, and IBA‐1 expression levels. Protocol details are provided in the Appendix S1.

### 2.5 Statistical analysis

Animal behavior during experiments was tracked with Anymaze software. Behavioral data from NORT, 3ct, and training phase of MWM were analyzed by three‐way ANOVA with repeated measures (fixed factors: sex, genotype, treatment, and within‐subjects factors: new/old object, new/old mouse/empty cage or day/trial, respectively) followed by pairwise comparisons with Sidak post hoc test. For repeated measures ANOVA, Greenhouse–Geisser correction was applied. Other behavioral data underwent three‐way ANOVA (for factors sex, genotype, and treatment) with Sidak post hoc test. All data were tested for normal distribution with Shapiro–Wilk test, but some groups did not meet normal distribution. As the sample size per group was relatively small, data transformation could not have changed the data distribution. It is demonstrated that ANOVA yields substantial robustness when applied on the data without normal distribution.^33^ Moreover, as there is no non‐parametric equivalent for three‐way ANOVA, we used this test for the analysis, but for the groups that failed to follow normal distribution, the non‐parametric Mann–Whitney U test was utilized to confirm those differences obtained after Sidak post hoc test between groups where at least one of groups did not follow normal distribution (cf. Ref. [34]). Out of 19 differences for the analyzed parameters revealed between two groups where at least one of them did not follow normal distribution, the non‐parametric testing confirmed significant differences in 14 cases, in 4 cases the difference was at a non‐significant statistical trend level (0.05 < *p* < 0.1), and only in 1 case, the significant difference disappeared; the *p* values are provided in Table S2 in Appendix S1. Accordingly, we decided to present and discuss all changes revealed by ANOVA testing, with the single exception of the case where non‐parametric analysis failed to demonstrate even a trend level of difference.

Statistical analysis was run in IBM SPSS Statistics 25 software and graphs were plotted in GraphPad Prism 9. The statistical significances are shown on graphs as * for 0.01 < *p* < 0.05, ** for 0.001 < *p* < 0.01, *** for *p* < 0.001. If significant, overall effects were reported in each graph.

## 3 RESULTS

### 3.1 Control transgenic females had impaired object memory and α5 GABA_A_ ligands were devoid of any effects on object memory in AD model

**FIGURE 2:**
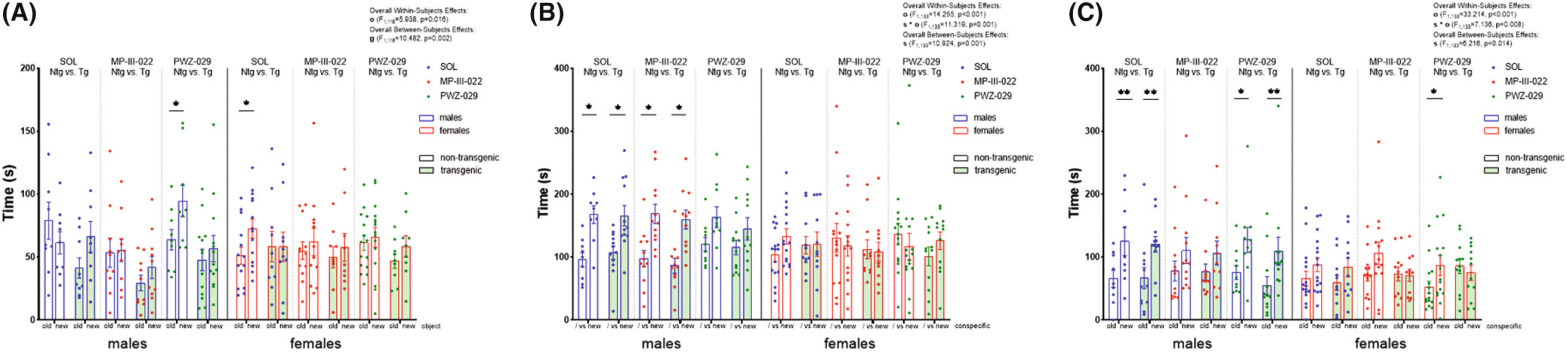
Results from the novel object recognition test for short‐term memory (NORT short‐term), social interaction test (SIT), and social recognition test (SRT) are shown for transgenic and non‐transgenic 5xFAD animals of both sexes treated with PWZ‐029, MP‐III‐022 or solvent. The time spent with the new vs old object for NORT short‐term, the time spent with empty cage vs cage with conspecific in SIT, and the time spent with cages with new vs old mouse in SRT are analyzed (A, B, and C, respectively). The statistical significances are shown on graphs as * for 0.01 < *p* < 0.05, ** for 0.001 < *p* < 0.01, *** for *p* < 0.001. The three‐way ANOVA with repeated measures and post hoc test Sidak were conducted for each experimental set of results. The overall effects if significant are given in the upper right corner. The abbreviations used are: g, s, t, and o for genotype, sex, treatment, and object (non‐social and/or social), respectively

Results and significant overall effects of factors genotype, treatment, sex or object or their interaction in NORT were shown in Figure 2.

In NORT for short‐term memory estimation, solvent‐treated non‐transgenic, but not transgenic females spent more time with a new compared to an old object (*p* = 0.045 and *p* = 0.985, respectively, Figure 2A). In males, neither solvent‐treated non‐transgenic nor transgenic animals did discriminate between the new and the old object (*p* = 0.186 and *p* = 0.251, respectively, Figure 2A). However, after PWZ‐029 administration, non‐transgenic males preferentially explored the new as compared to the old object (*p* = 0.022, Figure 2A). Neither PAM nor NAM treatment did have any effect in transgenic animals in NORT (*p* = 0.325 for males and *p* = 0.560 for females, and *p* = 0.416 for males and *p* = 0.379 for females, respectively, Figure 2A). In NORT for long‐term memory assessment, only non‐transgenic female controls showed a trend in favor of spending more time with the new as compared to the old object (*p* = 0.053, Figure S1 in Appendix S1).

### 3.2 MP‐III‐022 and PWZ‐029 impaired social recognition and social interaction, respectively, in 5xFAD mice

For 3ct, results and overall effects of factors genotype, treatment, sex or chamber or their interaction were included in Figure 2.

In SIT and SRT, both transgenic and non‐transgenic males treated with solvent spent more time in the chamber that contained a single unfamiliar animal or new animal, respectively, compared to chamber with empty cage or old animal, respectively (*p* = 0.036 and *p* = 0.028, respectively, Figure 2B). MP‐III‐022‐treated transgenic and non‐transgenic males explored more cage with conspecific compared to empty cage in SIT (*p* = 0.013 and *p* = 0.012, respectively, Figure 2B). On the other hand, PWZ‐029‐treated transgenic and non‐transgenic males explored more chamber with new conspecific, as compared to old in SRT (*p* = 0.005 and *p* = 0.015, respectively, Figure 2c). In SRT, PWZ‐029‐treated non‐transgenic females investigated more chamber with new conspecific compared to chamber with old conspecific (*p* = 0.046, Figure 2c).

### 3.3 MP‐III‐022 treatment impaired procedural learning in transgenic females, and transgenic females showed cognitive inflexibility regardless of treatment

**FIGURE 3:**
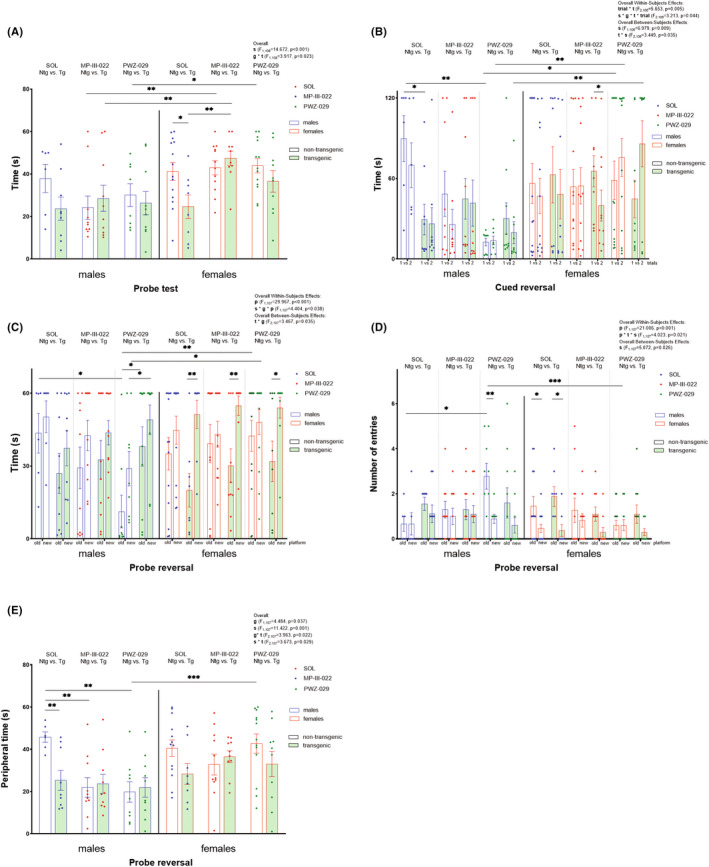
Results from the Morris water maze are selected for transgenic and non‐transgenic 5xFAD animals of both sexes treated with PWZ‐029, MP‐III‐022, or solvent. The time spent in peripheral zone (A) was measured for probe test. The latencies to reach the new platform in trials 1 vs 2 in reversal cued (B) were analyzed. In reversal probe, the latency to find new platform zone vs old platform zone (C), the number of entries in the new vs old platform zone (D) and the time spent in peripheral zone (E) were shown. The three‐way ANOVA with (B, C, D) or without (A, E) repeated measures followed by Sidak post hoc test was used. The statistical significances are shown on graphs as * for 0.01 < *p* < 0.05, ** for 0.001 < *p* < 0.01, *** for *p* < 0.001. The overall effects if significant are given in the upper right corner. The abbreviations used are: g, s, t, and *p* for genotype, sex, treatment, and platform, respectively

Results obtained in MWM, together with overall effects of factors genotype, treatment, sex or day (where applicable) or their interactions, were illustrated in Table 1 and Figure 3.

**TABLE 1:** Mean and standard error of latency to find the platform during regular Morris water maze (MWM) training

Sex	Genotype	Treatment	Mean of latency (s)	Standard error	*p* value (for all pairwise comparisons)
Males	Ntg	SOL	67.385	12.606	n.s.
		MP‐III‐022	49.902	9.765	n.s.
		PWZ‐029	53.711	10.917	n.s.
	Tg	SOL	49.603	10.293	n.s.
		MP‐III‐022	59.683	9.765	n.s.
		PWZ‐029	48.461	9.765	n.s.
Females	Ntg	SOL	66.955	8.253	n.s.
		MP‐III‐022	72.423	8.914	n.s.
		PWZ‐029	71.899	8.564	n.s.
	Tg	SOL	52.234 (for day 3: 44.438)	10.917 (for day 3: 12.823)	Tg females treated with MP‐III‐022 vs SOL (overall) *p* = 0.025 * (Tg females treated with MP‐III‐022 vs SOL for day 3: *p* = 0.024 *)
		MP‐III‐022	85.544 (for day 3: 90.955)	9.765 (for day 3: 11.469)	
		PWZ‐029	71.714	9.765	n.s.

*Note*: The significant results from the MWM are stated for transgenic and non‐transgenic 5xFAD animals of both sexes treated with PWZ‐029, MP‐III‐022, or solvent during whole training procedure. Additionally, any significant difference found on the each training was reported. Hence only transgenic female animals treated with MP‐III‐022 or SOL showed differences across all training days and on the training day 3, mean value and standard error for selected latencies were embedded within table. The three‐way ANOVA with repeated measures followed by Sidak post hoc test was utilized. The statistical significances are shown in the table as * for 0.01 < *p* < 0.05.

Transgenic females treated with MP‐III‐022 had higher latency to find platform compared to solvent‐treated transgenic females (*p* = 0.024, Table 1) at day 3 in regular MWM training. No significant difference between latencies of any other transgenic and non‐transgenic experimental groups across training days in regular MWM was revealed (Table 1). No significant difference for the latency to find the platform zone was found between groups in the probe day (Figure S2).

In probe trial, transgenic female controls spent less time in the periphery compared to non‐transgenic female controls (*p* = 0.016, Figure 3A). MP‐III‐022‐treated transgenic females spent more time in the peripheral zone compared to transgenic females treated with solvent (*p* = 0.007, Figure 3A).

In the reversal cued day, transgenic female animals treated with MP‐III‐022 had lower latencies to find the new platform in trial 2 compared to trial 1 (*p* = 0.031, Figure 3B). Further, transgenic male controls showed lower latency to find the new platform in trial 1 compared to control non‐transgenic male mice (*p* = 0.013, Figure 3B). Non‐transgenic male mice treated with PWZ‐029 had lower latency to find a new platform in trial 1 compared to control non‐transgenic males (*p* = 0.006, Figure 3B).

In reversal probe, transgenic females treated with MP‐III‐022, PWZ‐029, and solvent had higher latencies to find the new platform zone compared to old platform zone (*p* = 0.005, *p* = 0.012, and *p* = 0.002, respectively, Figure 3c). Furthermore, PWZ‐029‐treated non‐transgenic males had lower latency to find the old platform zone compared to non‐transgenic males treated with solvent (*p* = 0.036, Figure 3c). Both, transgenic and non‐transgenic solvent‐treated females had lower number of entries in the new platform zone compared to the old one (*p* = 0.011 and *p* = 0.029, respectively, Figure 3d). Similarly, PWZ‐029‐treated non‐transgenic males showed higher number of entries in old compared to the new platform zone (*p* = 0.001, Figure 3d), and also for the old platform zone, compared to solvent‐treated non‐transgenic males (*p* = 0.014, Figure 3d). Furthermore, transgenic males treated with solvent spent less time in the periphery compared to non‐transgenic male controls (*p* = 0.009, Figure 3E). Non‐transgenic males treated with PWZ‐029 and MP‐III‐022 likewise spent less time in the periphery of the tank compared to non‐transgenic males treated with solvent (*p* = 0.003 and *p* = 0.006, respectively, Figure 3E).

### 3.4 Neuroinflammation in transgenic animals and protective effects of PWZ‐029 treatment

**FIGURE 4:**
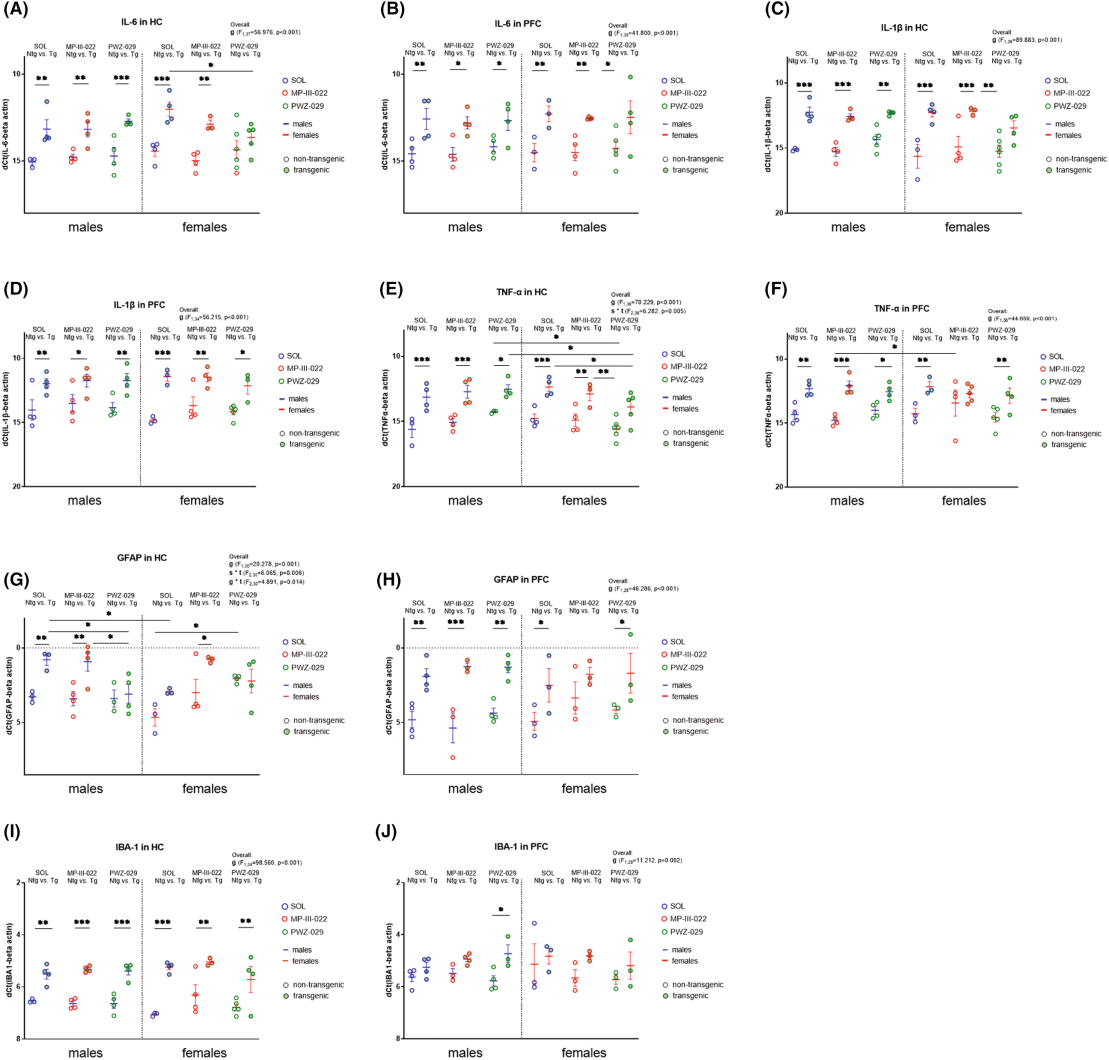
QPCR results from transgenic and non‐transgenic 5xFAD animals of both sexes treated with PWZ‐029, MP‐III‐022, or solvent. Gene expression for IL‐6, IL‐1β, TNF‐α as proinflammatory cytokines in hippocampus (HC) and prefrontal cortex (PFC) are shown (a‐f, respectively). The gene expression of GFAP and IBA‐1 as markers of astrogliosis and microgliosis, respectively, were determined in HC and PFC (g‐j, respectively). The statistical significances were revealed with the three‐way ANOVA followed by Sidak post hoc test and are shown on graphs as * for 0.01 < *p* < 0.05, ** for 0.001 < *p* < 0.01, *** for *p* < 0.001. The overall effects if significant are given in the upper right corner. The abbreviations used are: g, s, and t for genotype, sex, and treatment, respectively

Results from qPCR experiment and overall effects of factors genotype, treatment, and sex or their interactions are shown in Figure 4.

In HC and PFC, levels for IL‐6 (*p* = 0.004 and *p* < 0.001, respectively in HC, Figure 4A; *p* = 0.007 and *p* = 0.009 in PFC, respectively, Figure 4B), IL‐1β (*p* < 0.001 and *p* < 0.001 in HC, respectively, Figure 4c; *p* = 0.007 and *p* < 0.001 in PFC, respectively, Figure 4d), and TNF‐α (*p* = 0.001 and *p* < 0.001 in HC, respectively, Figure 4E; *p* = 0.004 and *p* = 0.008 in PFC, respectively, Figure 4F) were higher in male and female transgenic animals treated with solvent compared to male and female non‐transgenic controls, respectively. In HC, transgenic females treated with PWZ‐029 showed decreased transcript levels for IL‐6 (*p* = 0.015, Figure 4A) and TNF‐α (*p* = 0.033, Figure 4E) compared to solvent‐treated transgenic females.

GFAP levels in HC in transgenic male controls were higher compared to non‐transgenic male control (*p* = 0.008, Figure 4G), while transgenic female controls showed a trend effect toward increasing GFAP levels in HC compared to female non‐transgenic controls (*p* = 0.058, Figure 4G). Additionally, transgenic male controls had higher GFAP levels compared to transgenic female control in HC (*p* = 0.021, Figure 4G). PWZ‐029‐treated transgenic males and non‐transgenic females showed lower and higher GFAP levels compared to solvent‐treated transgenic males (*p* = 0.026, Figure 4G) and non‐transgenic females (*p* = 0.011, Figure 4G), respectively, in HC. In PFC, both male and female transgenic animals treated with solvent had higher GFAP levels compared to non‐transgenic control males (*p* = 0.003, Figure 4H) and females (*p* = 0.027, Figure 4H), respectively. IBA‐1 levels were higher in male and female transgenic controls compared to male (*p* = 0.004, Figure 4i) and female non‐transgenic controls (*p* < 0.001, Figure 4i), respectively, in HC. No statistical difference for IBA‐1 levels in PFC was found between groups (Figure 4J).

## 4 DISCUSSION

The current study in 5xFAD mice aimed to model early AD‐like changes in different cognitive domains and sociability. It assessed the consequences of protracted α5GABA_A_ receptor positive or negative modulation, elicited by selective ligands MP‐III‐022^27^ and PWZ‐029,^26^ respectively.

Performance in the novel object recognition task for short‐term object memory was impaired in transgenic compared to non‐transgenic 6‐month‐old female animals, providing the evidence that the model was successful to mimic object memory decline, as previously reported.^35^ Not only that MP‐III‐022 failed to demonstrate improvement in object memory in transgenic female animals, but also suppressed this type of memory in non‐transgenic females. These data are aligned with results obtained in rats, with MP‐III‐022 suppressing long‐term object memory when dosed at 10 mg/kg 24 h before the test.^9^

On the other hand, not only control transgenic, but also non‐transgenic males did not discriminate between new and old objects, which could be the result of sex‐dependent differences in object recognition, with female mice expected to be superior if the novel object was similar to a previously learned one.^36^ Both MP‐III‐022 and PWZ‐029 did not have any effect on object memory in transgenic males. In line with previous studies showing NAM could improve object memory (e.g., Ref. [12]), PWZ‐029 administration enhanced object memory in non‐transgenic males, implying a sex‐dependent procognitive effect.

Cortical GABA_A_ receptors exert a role in regulation of sociability,^15^ and thus we wanted to investigate if modulation of α5 GABA_A_ receptors could affect social interaction and/or social memory. In fact, the social interaction estimated in transgenic animal models related to amyloid and tau pathologies was shown to be reduced (for review see^37^). In accordance, animals from 5xFAD model which are in more advanced age, exhibit social withdrawal linked to progressive AD‐related pathology.^37^ As expected,^38^ in our study with animals in less advanced age that corresponds to the early (initiating) changes in behavioral outputs, both transgenic and non‐transgenic male animals still preferred social interaction over exploration of an empty cage. PWZ‐029 treatment impaired social interaction in both transgenic and non‐transgenic males. These results are in partial discordance with findings that selective α5 GABA_A_ receptor inverse or partial agonists did not affect sociability in Sprague–Dawley rats, while FG7142, nonselective inverse agonist at the GABA_A_ receptor, reduced social interaction.^16^ On the other hand, although prosocial effects were found after stereotaxic administration of the GABA_A_ receptor agonist muscimol in rats,^39^ MP‐III‐022 did not have any influence on social interaction of transgenic and non‐transgenic males in the present study.

As likewise expected,^38^ social memory was preserved in transgenic and non‐transgenic male controls, while MP‐III‐022 induced social memory impairment in males of both genotypes. The latter results are consistent with findings of impaired social discrimination elicited by MP‐III‐022 treatment in Wistar rats.^9^ PWZ‐029 did not have any influence, which is in line with research by Paine and co‐workers.^16^

Transgenic and non‐transgenic females failed to demonstrate normal social interaction and social memory. After PWZ‐029, non‐transgenic females were able to distinguish between the new and old mouse in chambers. In order to avoid any violations of pre‐determined timeline of the behavioral protocol, the estrus cycle stage of females was not determined, and this could be a possible reason for the lack of normal social interaction in control females.^40^ Another contributing factor may be the fact that female rodents tend to explore environment more than males, which may result in less interaction.^41^

Control transgenic and non‐transgenic animals showed comparable memory during training sessions in MWM as well as on the probe day, as previously reported.^42^ The finding could stem from suboptimal learning abilities of the tested mice due to retinal degeneration gene inherited from SJL background, as previously described.^43^

MP‐III‐022 treatment in transgenic females appeared to be detrimental on learning in the training phase of MWM, which is expected for positive modulation of α5 GABA_A_ receptors.^44^ In the first day of cued reversal learning phase, MP‐III‐022 treated transgenic females were enabled to find the new platform across trials. Non‐transgenic male animals treated with PWZ‐029 had the shortest latency for finding the new platform in reversal training day 1 compared to solvent‐treated non‐transgenic males, which points to a successful previous learning as a beneficial consequence of negative modulation of α5 GABA_A_ receptors.

In reversal probe, transgenic females, independently of treatment, showed higher latencies to find the new platform compared to old one, which could indicate a sex‐dependent impaired cognitive flexibility as reported in different protocols for the reversal probe for 3‐month‐old 5xFAD males^45^ and 3‐15‐month old 5xFAD animals of both sexes.^46^ PWZ‐029 vs. control‐treated non‐transgenic males showed an enhanced exploration of the old platform, as reflected in the number of entries in the old platform zone and latency to find the old platform, which could be an indicator of cognitive perseverance.^47^

Even though PWZ‐029‐treated non‐transgenic males showed a similar pattern in the number of entries in the old vs new platform zone as solvent‐treated transgenic females, there were differences in animals' ability to find the new platform in the latter group. Although the overall higher preference for the old platform could be the consequence of protocol design (as reversal learning was shorter than regular learning, and memory extinction could not be reached), memory consolidation is still assumed to have happened. Based on this hypothesis, reversal probe task would be successful if the animal swam in both platform zones.

In transgenic females, MP‐III‐022 treatment led to an increased peripheral time in the probe test and thus probably had a negative impact on procedural component of acquisition, as expected for positive modulation of α5 GABA_A_ receptors.^44^ On the other hand, transgenic male and female controls, in line with their behavior in the elevated plus maze and open field,^28^ had decreased emotional reactivity, manifested as less time spent in the thigmotaxic zone compared to their healthy controls in reversal probe and probe test, respectively. However, both ligand treatments, compared to solvent, were apparently linked with decreased anxiety in reversal probe in non‐transgenic males, as reflected in lower peripheral time.

Recent meta‐analysis did not confirm any increase of proinflammatory cytokines in blood of AD patients without depressive symptoms,^48^ but this does not address possible local neuroinflammation. Up‐regulation of GFAP mRNA in PFC and HC, observed in transgenic compared to non‐transgenic mice, demonstrates an increased astrogliosis, and is concordant with increased expression of GFAP protein in cortex and hippocampus of 5xFAD mice.^35^ Intriguingly, GFAP levels in transgenic male controls were higher compared to transgenic female controls. In HC, after PWZ‐029 administration, GFAP transcript levels in transgenic males were decreased compared to solvent‐treated transgenic males, while GFAP levels in non‐transgenic females were increased compared to solvent‐treated ones.

In 5xFAD transgenic model, pathology‐related microglial phenotype is found, and results are translated to human microglia in AD.^49^ In our study, microgliosis was demonstrated by increased IBA‐1 expression in HC of solvent‐treated transgenic animals, as compared to non‐transgenic animals regardless of sex influence; in PFC, such a difference was not detected despite an overall genotype effect.

Interleukin‐1 (IL‐1), interleukin‐6 (IL‐6), and tumor necrosis factor α (TNF‐α) are produced mainly by immune cells, but could also originate from neural cells.^50^, ^51^ In our study, all proinflammatory cytokine transcripts were upregulated in transgenic animals compared to non‐transgenic animals regardless of sex. IL‐1 could contribute to synaptic loss and TNF‐α could induce cell death,^19^ suggesting their significant interplay in chronic inflammatory processes such as AD. Administration of IL‐1 receptor antagonist antibody in an AD mouse model restored cognitive deficits observed in Morris water maze and reduced neuroinflammation.^52^ IL‐6 was (or at least tended to be) up‐regulated in PFC and HC of AD patients.^53^, ^54^ In a rat AD model, IL‐1β, IL‐6, and TNF‐α were increased in the hippocampus.^52^, ^55^

Recently, GABA caught attention as an important signaling mediator in inflammatory coordination. It was discovered that GABA from B lymphocytes could inhibit CD8+ T cell killer response, and blockade of GABA production in B lymphocytes resulted in higher anti‐tumor response.^56^ Here, we detected that NAM could influence neuroinflammation in hippocampus, as previous treatment with PWZ‐029 decreased inflammation in transgenic female hippocampus compared to their transgenic control. Furthermore, NAM downregulated GFAP in transgenic males, and upregulated GFAP in non‐transgenic females compared to their controls. These paradoxically opposite effects suggest sex and genotype dependence of negative allosteric modulation of α5 GABAA receptors. Behavioral relevance of these effects could not be addressed in this study and further research is needed.

It could be generally hypothesized that bidirectional pharmacological modulation of a distinct target, putatively involved in initiation and/or development of a pathological process, should result in opposite effects of two tested ligands. If, tentatively speculated, the selected target is pathologically decreased or suppressed, its pharmacological potentiation (i.e., positive modulation) and further inhibition (i.e., negative modulation) would be expected to normalize and further deteriorate the assessed biological parameter, respectively. However, there are two major factors that limit the experimental detection of such theoretical considerations, especially in terms of behavioral parameters. First, the challenge of existence of the flooring and ceiling effect, both of which not only depend on the level of difficulty of a test, but are also easily encountered while testing in parallel the impaired and normally functioning animals.^57^ Second, the complexity and degeneracy of neural pathways controlling neurophysiology and behavior,^58^ which means that the obtained degree of activity of a single, highly specific target, although impacting distinct aspects of neural plasticity, still does not necessarily shift the measured biological and behavioral outcomes. Indeed, it seems more probable to reveal opposite effects if more targets are involved, e.g. by use of non‐selective positive and negative allosteric modulators, such as clobazam and DMCM. In this vein, single low dose of clobazam ameliorated social interaction deficits in autistic‐like BTBR mice, while DMCM reduced normal social interaction behavior in both C57BL/6J and 129SvJ mice.^59^ Nonetheless, it is notable that different subtypes of GABA_A_ receptors may have opposite roles in social behavior, with activation of GABA_A_ receptors containing α2 or α3 subunits favoring, but activation of GABA_A_ receptors with α1 subunits reducing social interaction, respectively.^59^ To add to the conundrum, even the unidirectional non‐selective modulation of GABA_A_ receptors may result in opposite effect dependent on the strain/health status of animals used: thus, non‐selective positive modulation with clonazepam significantly improved the spatial learning performance of BTBR mice, while worsened the performance of control C57BL/6J mice.^59^ Finally, the literature offers evidence that similar behavioral outputs, related to diminished nociceptive behavior in rat models of inflammatory or neuropathic pain, may be induced by administration of PAMs with predominant activity at GABA_A_ receptors that contain the α5, α3, and α2 subunits, but also by NAMs that act at α5 GABA_A_ receptors, or even are non‐selective in their activity (Munro et al., 2011).

All aforementioned evidence indicates that the lack of clear‐cut opposite effects of the selected PAM and NAM tested in this and previous study^28^ was far from unexpected. Moreover, the PAM and NAM effects were strongly dependent on the experimental group, and could be similar, opposite, or neutral. Additionally,in line with our results, there is evidence from experiments with GABA_A_ modulators that, besides genetic background (cf. ^59^), sex also impactsligand effects on certain molecular and behavioral domains (cf. ^60^, ^61^). Taken together, PAM treatment in our study had detrimental effect on social recognition in transgenic and non‐transgenic males, and on spatial learning in transgenic females. To the contrary, it potentiated cognitive flexibility in transgenic females. NAM treatment could not alleviate memory in transgenic animals, but boosted social recognition and spatial cognition in reversal phase in non‐transgenic females and non‐transgenic males, respectively. NAM treatment also gave rise to a decline of social interaction in transgenic and non‐transgenic males. Finally, NAM reduced inflammation in female transgenic hippocampus and astrogliosis in male transgenic hippocampus.

In conclusion, in 6‐month‐old mice of 5xFAD AD model, we identified object memory decline and neuroinflammation in both sexes, and cognitive inflexibility in females. PAM improved memory flexibility, while NAM reduced neuroinflammation. However, PAM and NAM worsened pathology in spatial and social memory, and sociability domains, respectively. The results obtained in previous research, related to PAM and NAM beneficial effects on different types of memory impairment induced in healthy animals, should be interpreted with caution in the context of AD, as a complex multifactorial pathology that involves chronic neuroinflammation.

## CONFLICT OF INTEREST

The authors have no conflict of interest to report.

## Supporting information


Appendix S1

## Data Availability

The authors confirm that the data supporting the findings of this study are available within the article and its supplementary materials. Raw data are available from the corresponding author [Miroslav M. Savić] upon reasonable request.
