# Supplementary material for: Effects of α5 GABAA receptor modulation on social interaction, memory, and neuroinflammation in a mouse model of Alzheimer's disease
Source: CNS Neurosci Ther. 2022 Jul 13;28(11):1767–78. doi: 10.1111/cns.13914 (PMC9532908; doi:10.1111/cns.13914)
Supplement: Supplementary file 1 — Appendix S1 [file CNS-28-1767-s001.docx]

**Supplementary Material for the manuscript:**

**α5 GABAA receptor modulation effects on social interaction, memory and neuroinflammation in mouse model of Alzheimer’s disease**

Jovana Aranđelović^a^, Anja Santrač^a^, Bojan Batinić^b^, Vladimir Stevanović^a^, Lidija Todorović^c^, Veera Venkata Naga Phani Babu Tiruveedhula^d^, Dishary Sharmin^d^, Farjana Rashid^d^, Boban Stanojević^c,e^, James M. Cook^d^, Miroslav M. Savić^a^

^a^Department of Pharmacology, Faculty of Pharmacy, University of Belgrade, 450 Vojvode Stepe St, 11000, Belgrade, Serbia

^b^Department of Physiology, Faculty of Pharmacy, University of Belgrade, 450 Vojvode Stepe St, 11000, Belgrade, Serbia

^c^Laboratory for Radiobiology and Molecular Genetics, Vinča Institute of Nuclear Sciences, National Institute of thе Republic of Serbia, University of Belgrade, Belgrade, Serbia

^d^Department of Chemistry and Biochemistry, Milwaukee Institute for Drug Discovery, University of Wisconsin-Milwaukee, 3210 N. Cramer St., Milwaukee, Wisconsin 53201, USA

^e^Comprehensive Cancer Centre, Faculty of Life Sciences & Medicine, King’s College London, Rayne Institute, 111 Coldharbour Ln, London SE5 9RR, UK.

Table of Contents

**1. Supplementary Material and methodology 3**

**Subjects 3**

**RNA isolation, reverse transcription and qPCR 3**

**2. Supplementary Results and sample size 4**

**3. References 7**

**1.** **Material and methodology**

**Subjects**

Due to aggressiveness of male mice, a short plastic tunnel was introduced in each cage. Mice were thoroughly handled during 7 days before the beginning of behavioral tests, while physical observation and body mass measurement were carried out throughout the study.

RNA isolation, reverse transcription and qPCR

Total RNA isolation was performed using TRI Reagent solution (Ambion, Foster City, CA) and chloroform, according to the manufacturer’s instructions. RNA quantification was done on BioSpec Nano (Shimadzu, Japan). After RNA concentration measurement, 1 μg of RNA was converted into cDNA by High Capacity cDNA Reverse Transcription Kit (Applied Biosystems, Foster City, CA). The PCR reactions were performed on the 7500 Real Time PCR System (Applied Biosystems, Foster City, CA). PCR reactions were carried out in 10 μl reaction volume containing 5 μl Power SYBR® Green PCR Master Mix (Life Technologies, Warringtone, UK), 300 nM of each forward and reverse primer and cDNA corresponding to 10 ng of total RNA equivalent. The cycling conditions were: 10 min at 95°C, 40 cycles of 15 s at 95°C and 60 s at 60°C. A melting curve was generated for every PCR product to ensure the specificity of the reaction. Primers for IL-1β, IL-6, TNFα, IBA-1, GFAP and beta actin as the housekeeping gene were selected from the literature (F: GAAATGCCACCTTTTGACAGT and R: CTGGATGCTCTCATCAGGACA (Sayed et al., 2021), F: TAGTCCTTCCTACCCCAATTTCC and R: TTGGTCCTTAGCCACTCCTTC (Mathur et al., 2017), F: CTGAACTTCGGGGTGATCGG and R: GGCTTGTCACTCGAATTTTGAGA (Takano et al., 2016); F: GGATTTGCAGGGAGGAAAAG and R: TGGGATCATCGAGGAATTG (Kang et al., 2018), F: GGCGCTCAATGCTGGCTTCA and R: TCTGCCTCCAGCCTCAGGTT (Chandra et al., 2017), F: GACCTCTATGCCAACACAGT and R: AGTACTTGCGCTCAGGAGGA (Wang et al., 2019), respectively). Ct values were calculated using Applied Biosystems 7500 system SDS software. The dCt was calculated as the difference between Ct(selected gene) and Ct(beta actin) for each sample, and was inversely proportional to the mRNA expression.

The three-way ANOVA followed by Sidak post hoc test was applied to determine differences between groups.

**Results**

Novel object recognition test for long-term memory


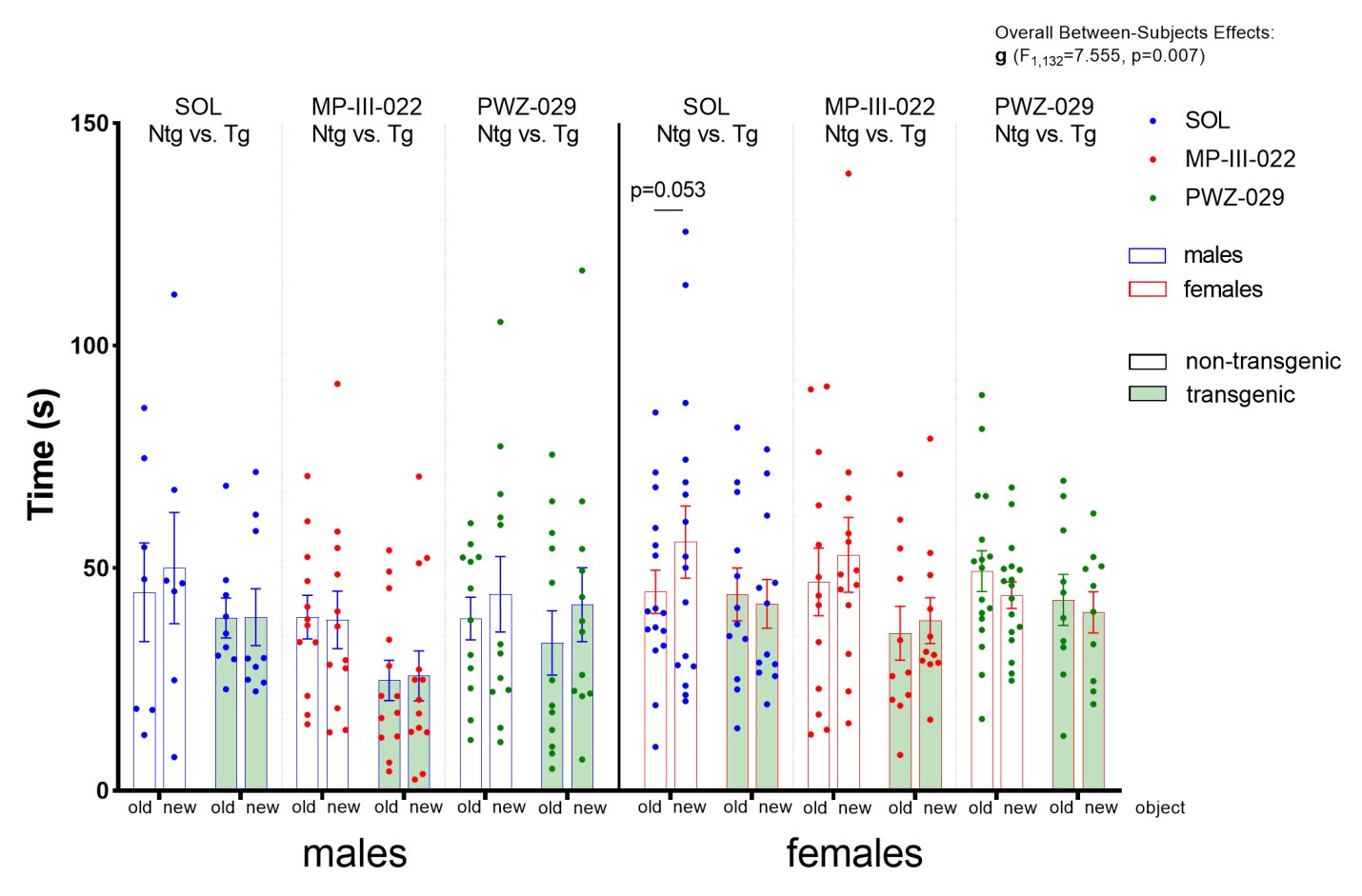


Figure S1. Results from the novel object recognition test for long-term memory (NORT long-term) are shown for transgenic and non-transgenic 5xFAD animals of both sexes treated with PWZ-029, MP-III-022 or solvent. The time spent with the new vs old object for NORT long-term was subjected to the three-way ANOVA with repeated measures followed by Sidak post hoc test. The statistical significances are shown on graph as * for 0.01<p<0.05, ** for 0.001<p<0.01, *** for p<0.001 if applicable. Non-significant statistical trend is written (0.05<p<0.1). The overall effects if significant are given in the upper left corner. The abbreviations used are: g, s, t and o for genotype, sex, treatment and object, respectively.

Probe test in Morris water maze (MWM) protocol


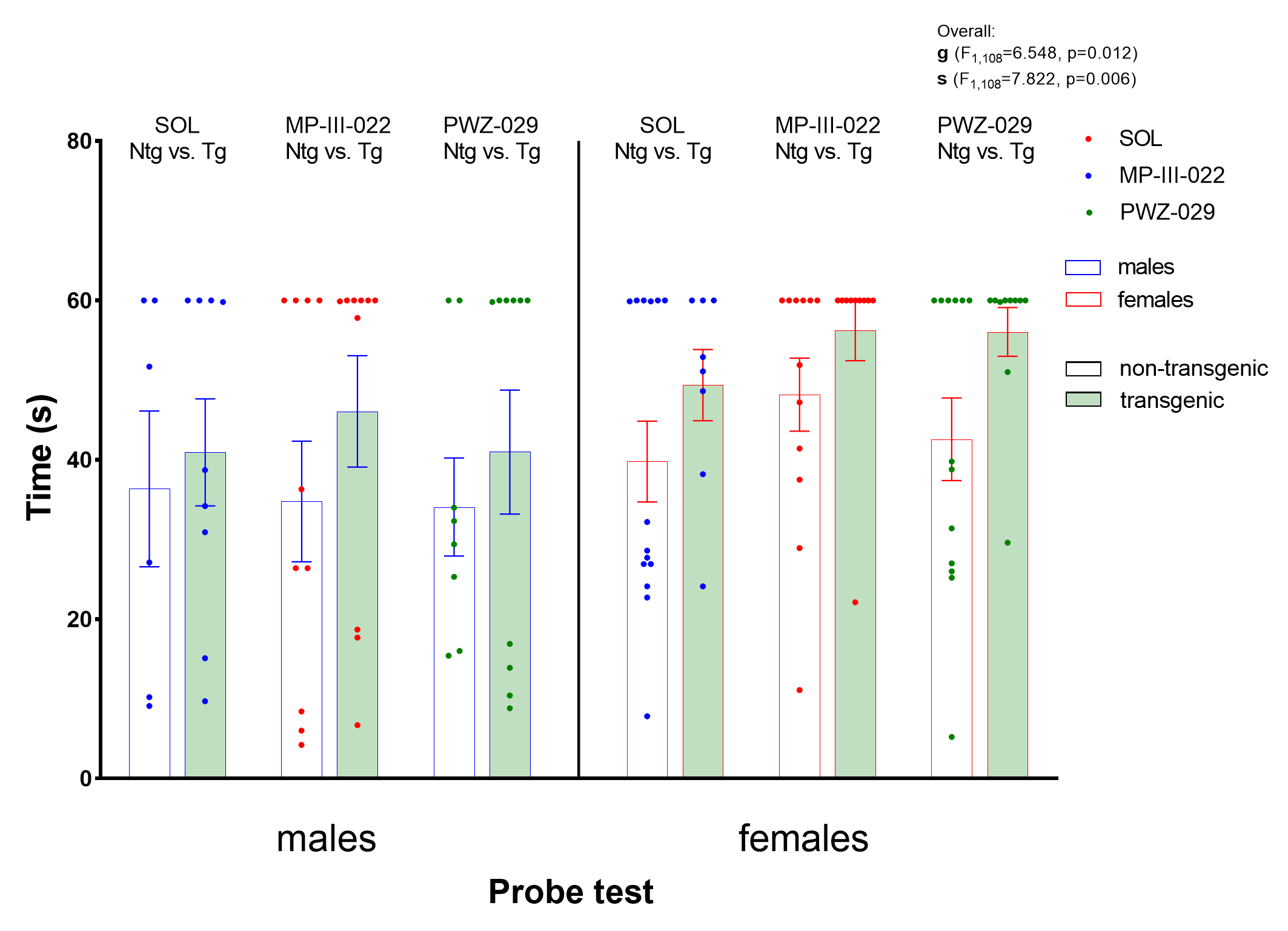


Figure S2. Results from the Morris water maze test (MWM) for spatial memory are shown for transgenic and non-transgenic 5xFAD animals of both sexes treated with PWZ-029, MP-III-022 or solvent. The results for the latency to reach platform zone were analyzed by the three-way ANOVA with repeated measures followed by Sidak post hoc test. The statistical significances are shown on graph as * for 0.01<p<0.05, ** for 0.001<p<0.01, *** for p<0.001 if applicable. The overall effects if significant are given in the upper left corner. The abbreviations used are: g, s, t and o for genotype, sex, treatment and object, respectively.

**Sample size**

Table S1. Number of animals per group across different experiments. Non-equal sample size in different tests within each group was a consequence of certain number of failures during Anymaze software tracking. Additionally, light-fur animals within the first two cohorts were not analyzed in MWM procedure due to technical difficulties. S – solvent, M – MP-III-022, P – PWZ-029

| **Number of animals per group** | **Non-transgenic males**  **(Ntgm)** | | | **Transgenic males**  **(Tgm)** | | | **Non-transgenic females**  **(Ntgf)** | | | **Transgenic**  **females (Tgf)** | | |
| --- | --- | --- | --- | --- | --- | --- | --- | --- | --- | --- | --- | --- |
|  | **S** | **M** | **P** | **S** | **M** | **P** | **S** | **M** | **P** | **S** | **M** | **P** |
| Nort short-term | 9 | 10 | 9 | 10 | 10 | 13 | 14 | 12 | 15 | 10 | 9 | 9 |
| Nort long-term | 7 | 12 | 12 | 9 | 13 | 12 | 16 | 13 | 17 | 12 | 11 | 10 |
| SIT | 9 | 12 | 10 | 12 | 11 | 12 | 16 | 14 | 15 | 11 | 11 | 12 |
| SRT | 9 | 12 | 10 | 12 | 11 | 12 | 16 | 14 | 15 | 11 | 11 | 12 |
| MWM training and probe | 6 | 10 | 8 | 9 | 10 | 10 | 14 | 12 | 13 | 8 | 10 | 10 |
| MWM reversal qued and reversal training | 6 | 10 | 9 | 9 | 10 | 10 | 14 | 12 | 13 | 8 | 10 | 10 |
| MWM reversal probe | 6 | 10 | 9 | 9 | 10 | 10 | 13 | 11 | 13 | 8 | 10 | 10 |
| GFAP HC | 3 | 4 | 3 | 3 | 4 | 4 | 3 | 4 | 4 | 3 | 3 | 4 |
| GFAP PFC | 4 | 3 | 4 | 4 | 3 | 4 | 3 | 3 | 3 | 3 | 3 | 3 |
| IBA-1 HC | 3 | 4 | 4 | 4 | 4 | 4 | 3 | 4 | 5 | 4 | 3 | 4 |
| IBA-1 PFC | 4 | 3 | 4 | 4 | 4 | 3 | 3 | 3 | 3 | 3 | 3 | 3 |
| IL-1β HC | 3 | 4 | 5 | 4 | 4 | 4 | 3 | 4 | 6 | 4 | 3 | 4 |
| IL-1β PFC | 4 | 4 | 4 | 4 | 4 | 4 | 3 | 4 | 5 | 3 | 4 | 3 |
| IL-6 HC | 3 | 4 | 4 | 4 | 4 | 4 | 4 | 4 | 6 | 4 | 3 | 5 |
| IL-6 PFC | 4 | 4 | 4 | 4 | 4 | 4 | 3 | 4 | 5 | 3 | 4 | 4 |
| TNFα HC | 3 | 4 | 3 | 4 | 4 | 4 | 4 | 4 | 6 | 4 | 3 | 5 |
| TNFα PFC | 4 | 4 | 4 | 4 | 4 | 4 | 3 | 4 | 5 | 3 | 5 | 4 |

The same three consecutively evaluated cohorts underwent behavioral tests as in Aranđelović et al. 2021.

Table S2: The p values revealed after parametric and non-parametric testing for selected parameters for certain groups, where at least one of the group data did not follow normal distribution. For parametric statistical analysis, three-way ANOVA with or without repeated measures followed by Sidak post hoc test, where applicable, were conducted, whereas non-parametric statistical analysis was performed with Mann Whitney test. The significant p values are reported without any label (p<0.05), non-significant statistical trends are underlined (0.1>p>0.05) and non-significant p values are labeled with ^ (p>0.1).

| **Experiment** | **Parameter** | **Treated groups** | **P value (after parametric testing)** | **p value (after non-parametric testing)** |
| --- | --- | --- | --- | --- |
| **SRT** | Time with new vs old animal | Ntg males SOL | 0.028 | p=0.0029 |
| **SRT** | Time with new vs old animal | Tg males PWZ-029 | p=0.005 | p=0.0083 |
| **SRT** | Time with new vs old animal | Ntg females PWZ-029 | p=0.046 | p=0.0613 |
| **MWM training** | Latency for day 3 | Tg females treated with  MP-III-022 vs SOL | p=0.024 | P=0.0115 |
| **MWM reversal qued training** | Latency to find new platform in trial 1 vs trial 2 | Tg females MP-III-022 | p=0.031 | p=0.0785 |
| **MWM reversal qued training** | Latency to find new platform in trial 1 vs trial 2 | Tg females PWZ-029 | p=0.001 | p=0.2241 ^ |
| **MWM reversal qued training** | Latency to find new platform in trial 1 vs trial 2 | Tg males SOL | p=0.013 | p=0.0164 |
| **MWM reversal qued training** | Latency to find old platform in trial 1 | Ntg males treated with PWZ-029 vs SOL | p=0.006 | p=0.001 |
| **MWM reversal probe** | Latency to find new vs old platform | Tg females MP-III-022 | p=0.005 | p=0.0126 |
| **MWM reversal probe** | Latency to find new vs old platform | Tg females PWZ-029 | p=0.012 | p=0.0409 |
| **MWM reversal probe** | Latency to find new vs old platform | Tg females SOL | p=0.002 | p=0.0118 |
| **MWM reversal probe** | Latency to find new vs old platform | Ntg males PWZ-029 | p=0.036 | p=0.0074 |
| **MWM reversal probe** | Number of entries in new vs old platform zone | Tg females SOL | p=0.011 | p=0.0177 |
| **MWM reversal probe** | Number of entries in new vs old platform zone | Ntg females SOL | p=0.029 | p=0.0656 |
| **MWM reversal probe** | Number of entries in new vs old platform zone | Ntg males PWZ-029 | p=0.001 | p=0.0125 |
| **MWM reversal probe** | Number of entries in old platform zone | Ntg males PWZ-029 vs SOL | p=0.014 | p=0.0214 |
| **MWM reversal probe** | Peripheral time (%) | Tg males SOL vs Ntg males SOL | p=0.009 | p=0.0082 |
| **qPCR** | IL-6 gene expression in hippocampus | Tg males SOL vs Ntg males SOL | p=0.004 | p=0.0571 |
| **qPCR** | IL-6 gene expression in prefrontal cortex | Tg males SOL vs Ntg males SOL | p=0.007 | p=0.0286 |

**References**

1. Aranđelović J, Santrač A, Batinić B, Todorović L, Ahmed Khan MZ, Rashid F, Poe MM, Obradović A, Cook JM, Savić MM. Positive and Negative Selective Allosteric Modulators of α5 GABAA Receptors: Effects on Emotionality, Motivation, and Motor Function in the 5xFAD Model of Alzheimer's Disease. J Alzheimers Dis. 2021;84(3):1291-1302. doi: 10.3233/JAD-215079. PMID: 34657887.
2. Chandra, G., Roy, A., Rangasamy, S. B., & Pahan, K. (2017). Induction of Adaptive Immunity Leads to Nigrostriatal Disease Progression in MPTP Mouse Model of Parkinson's Disease. Journal of immunology (Baltimore, Md. : 1950), 198(11), 4312–4326. <https://doi.org/10.4049/jimmunol.1700149>
3. Kang, S. S., Kurti, A., Baker, K. E., Liu, C. C., Colonna, M., Ulrich, J. D., Holtzman, D. M., Bu, G., & Fryer, J. D. (2018). Behavioral and transcriptomic analysis of Trem2-null mice: not all knockout mice are created equal. Human molecular genetics, 27(2), 211–223. <https://doi.org/10.1093/hmg/ddx366>
4. Mathur, V., Burai, R., Vest, R. T., Bonanno, L. N., Lehallier, B., Zardeneta, M. E., Mistry, K. N., Do, D., Marsh, S. E., Abud, E. M., Blurton-Jones, M., Li, L., Lashuel, H. A., & Wyss-Coray, T. (2017). Activation of the STING-Dependent Type I Interferon Response Reduces Microglial Reactivity and Neuroinflammation. *Neuron*, *96*(6), 1290–1302.e6. <https://doi.org/10.1016/j.neuron.2017.11.032>
5. Sayed, I. M., Ibeawuchi, S. R., Lie, D., Anandachar, M. S., Pranadinata, R., Raffatellu, M., & Das, S. (2021). The interaction of enteric bacterial effectors with the host engulfment pathway control innate immune responses. Gut microbes, 13(1), 1991776. <https://doi.org/10.1080/19490976.2021.1991776>
6. Takano, S., Uchida, K., Miyagi, M., Inoue, G., Fujimaki, H., Aikawa, J., Iwase, D., Minatani, A., Iwabuchi, K., & Takaso, M. (2016). Nerve Growth Factor Regulation by TNF-α and IL-1β in Synovial Macrophages and Fibroblasts in Osteoarthritic Mice. Journal of immunology research, 2016, 5706359. <https://doi.org/10.1155/2016/5706359>
7. Wang, H., Cui, J., Yang, C., Rosenblum, J. S., Zhang, Q., Song, Q., Pang, Y., Fang, F., Sun, M., Dmitriev, P., Gilbert, M. R., Eisenhofer, G., Pacak, K., & Zhuang, Z. (2019). A Transgenic Mouse Model of Pacak⁻Zhuang Syndrome with An Epas1 Gain-of-Function Mutation. Cancers, 11(5), 667. https://doi.org/10.3390/cancers11050667
